# Preserving and Preparing Anatomical Meterial

**Published:** 1859-01

**Authors:** W. G. Smull

**Affiliations:** Demonstrator of Anatomy Balt. Col. Dent'l Surgery.


### ARTICLE III.

### *Preserving and Preparing Anatomical Material.*

By W. G. SMULL, M. D.

*Editors Amer. Journal of Dental Science:*

As the study of anatomy is taught with as much thoroughness in the Baltimore College of Dental Surgery as it is in the medical colleges, I have thought that it might not be out of place to offer a few suggestions relative to the modes of preserving and preparing anatomical material. Such information, I am certain, will be acceptable to those students of practical anatomy who intend prosecuting the study at home during the interval of the lecture seasons, at a time when the heat of the summer adds greatly to the liability to decomposition of their subjects.

The design in preparing a subject for dissection is, firstly, to prevent decomposition, and thus to allow more time for careful dissection; and secondly, to distend the arteries so as to give them the form which they have in the living body, and also to better guide the unpracticed anatomist in exposing them. A hasty method is frequently adopted of combining arsenic or some other antiseptic with the "thick" injection, and thereby an attempt is made to accomplish both at one time. The objection to this process is, that the disinfectant does not reach the tissues deeper than at the beginning of the capillaries and the infiltration of the disinfectant through the entire tissues, is impossible. The most certain plan of securing a subject from decomposition is to throw in the disinfecting injection at first, and allow it to remain at least a day, and then without any disturbance of the apparatus, the heavy injection may be introduced.

The disinfectants ordinarily used are arsenic, nitrate of potash, corrosive sublimate, alcohol, creosote, chloride of zinc, and dilute nitric acid. The latter two are those to which I give decided preference, and deserve more than a passing notice. Aside from the objection of the former ones not being sufficiently efficacious as disinfectants, the use of them is attended with other undesirable results; the solution of arsenic is found deposited on the surface of the muscle as soon as it has been exposed a short time, while the solution of corrosive sublimate rapidly destroys the dissecting instruments. The solution of chloride of zinc will arrest decomposition for a great length of time, and when properly used, keeps the dissecting room free from unpleasant effluvia.

The formula which I generally use is

Chloride of zinc, 1 ounce.
Water, 1 gallon.

This is strong enough for ordinary purposes, but might be used stronger in the summer season. The objection, and the only one, to this preparation is, that it whitens the tissues, making the muscular fibre less distinct than might be desired. The formula for the nitric acid injection is

Nitric acid, lbi.
Water, five gallons.

Although this proves an excellent disinfectant and preserves the color of the muscles perfectly natural, it renders the bones unfit for preservation, the acid dissolving the earthy matter of the bones, and making them soft. Incipient decomposition may be soon arrested by sprinkling the part with this solution.

The formulæ for the permanent injection are numerous, and are usually made according to each one's favorite plan. Experiment shows great differences in the minuteness of the vessels injected, in the color and the firmness of the injection. Heavy injections, when thrown in cold, are apt either to set too quick or to remain fluid. I will give a few of the formulæ that I have found to answer best; they are used at a temperature of 100° Fahrenheit, they set quickly and show a fine arterial color through the thin coats of the smaller arteries.

Tallow, 2 lbs.
Beeswax, ½ lb.
Red lead, 1 ounce.
Vermilion, 1 drachm.
Linseed oil, 8 ounces.

The lead and vermilion are stirred in slowly while the mixture is almost at a boiling point; the whole is strained and used before it cools. Sometimes turpentine or turpentine varnish is substituted for the oil; the purpose of the last ingredient being to give sufficient fluidity to the injection; the proportions in these cases remaining the same. Where finer injections are desired, the following would be more suitable.

Tallow, 1 lb.

Wax, ¼ lb.

Red lead, ½ ounce.

Vermilion, 2 drachms.

Turpentine varnish.

Linseed oil, of each, 8 ounces.

It should be remembered that dissection should not be begun so soon after using this as after the former formula.

Veins are seldom injected in ordinary dissections, and when desired in making preparations, indigo blue, with a small quantity of black lead or ivory black, may be used in place of the red coloring ingredients.

W. G. SMULL,

*Demonstrator of Anatomy Balt. Col. Dent'l Surgery.*

